# *Amaranthus hybridus (Amaranthaceae)* prevents the detrimental effects of cyclophosphamide on ovarian function in Wistar rats: An experimental study

**DOI:** 10.18502/ijrm.v20i8.11754

**Published:** 2022-09-06

**Authors:** Pierre Watcho, Prechmy Carole Nsamou Ngueyong, Patrick Brice Defo Deeh, Georges Romeo Bonsou Fozin, Esther Ngadjui, Modeste Wankeu-Nya, Pierre Kamtchouing

**Affiliations:** ^1^Research Unit of Animal Physiology and Phytopharmacology (URPAP), Faculty of Science, University of Dschang, Dschang, Cameroon.; ^2^Department of Biomedical Sciences, Faculty of Medicine and Pharmaceutical Sciences, University of Dschang, Dschang, Cameroon.; ^3^Department of Animal Organisms Biology, University of Douala, Douala, Cameroon.; ^4^Department of Animal Biology and Physiology, University of Yaoundé 1, Yaoundé, Cameroon.

**Keywords:** Cyclophosphamide, Amaranthus hybridus, Toxicity, Estradiol, Rat.

## Abstract

**Background:**

Cyclophosphamide (CP) is an anticancer agent, but its chronic administration induces ovarian toxicity.

**Objective:**

We evaluated the effects of aqueous extract (AE) and methanol extract (ME) of *Amaranthus hybridus* (*A.* *hybridus*) on CP-induced ovarian toxicity in rats.

**Materials and Methods:**

40 female Wistar rats (10 wk, 170-200 gr) were distributed into 8 groups (n = 5/each) as follows: 1) healthy control; 2) CP+distilled water (10 ml/kg/d); 3) CP+3%-tween 80 (10 mL/kg/d); 4) CP+clomiphene citrate (2 mg/kg/d); 5, 6) CP+AE of *A. hybridus *(55 and 110 mg/kg/d); and 7, 8) CP+ME of *A. hybridus *(55 and 110 mg/kg/d). After 28 days of treatment, estrus cyclicity, ovarian and uterine weights as well as estradiol levels and ovarian histology were determined.

**Results:**

CP induced ovarian toxicity after 28 days of exposure. More specifically, CP disturbed the estrus cycle, decreased ovary and uterus weights (p = 0.04), and the 17-β estradiol level (p = 0.04), and induced severe ovarian damages. Remarkably, *A. hybridus *significantly increased (p = 0.03) the ovarian weight (AE and ME at all doses) and uterus weight (ME at 110 mg/kg/d), compared with the CP-treated rats. Moreover, the 17-β estradiol level was significantly elevated (p = 0.02) in rats given clomiphene citrate and *A. hybridus *(AE 110 mg/kg/d; ME 55 mg/kg/d). Finally, the ovaries of rats given plant extracts had many corpus luteum and normal follicles, and no cystic follicles.

**Conclusion:**

prevented the detrimental effects of CP on ovarian function, which could support its traditional use as a fertility enhancer.

## 1. Introduction

Cyclophosphamide (CP) is a cytotoxic agent commonly used to treat several types of cancers in children, adolescents, and adults (1). However, this chemotherapy negatively affects the female reproductive system and fertility, especially in younger patients under long-term CP treatment (2, 3). Chronic administration of CP alters the primordial follicles (ovarian reserve) and damages the growing ovarian follicles through a double mechanism. Firstly, by interacting with DNA and stimulating cellular apoptosis and cell death (4), it leads to the destruction of ovarian reserve follicles and inhibition of follicular maturation (5). CP also induces an overproduction of reactive oxygen species (ROS), which react with lipids, proteins, and cellular nucleic acids and inhibit steroidogenesis in ovarian cells, causing infertility (4). This reproductive toxicity of CP is mainly attributed to acrolein, an active metabolite of CP responsible for its side effects (5).

In female rats, reproductive toxicity is characterized by estrus cycle disruption, ovarian atrophy, presence of multiple cystic follicles, low 17-β estradiol (E2) concentration in the follicles as well as ovarian ultrastructure lesions (6). Modern treatment of reproductive toxicity involves the use of various drugs such as antioxidant agents and ovulation inductors, but it is commonly associated with severe side effects (7). Medicinal plants are extensively used in the treatment of reproductive toxicity, due to their biosafety, availability, and rich diversity of phytochemical compounds. Previous works have reported the efficacy of some herbal remedies on the prevention/treatment of CP-induced reproductive toxicity in female rats. For instance, the preventive effects of *Spirulina sp. *(8) and *Nigella sativa* (9) on CP-induced ovarian damages have been reported. In addition, curcumin (10) and Bushen Huoxue recipe (11) can alleviate the detrimental effects of CP on ovarian function by improving sex hormone levels, antioxidant enzymes, and ovarian reserve.

*Amaranthus hybridus* (*A.* *hybridus*) is a tropical plant commonly used as a food in the culinary arts and as a drug in folk medicine. In Cameroon, its leaves and seeds are used as fertility booster but no scientific report on this property has been published. Various phyto-components such as tannins, phenols, alkaloids, saponins, steroids, and triterpenes have been identified in this plant (12). Previous experiment studies revealed the antimicrobial (12), antidiabetic (13), anticancer (14) and antioxidant (15) potentials of *A. hybridus. *Despite the traditional use of* A. hybridus *to improve various aspects of reproduction, no scientific studies on the effects of this plant on female reproductive toxicity are available. This study was undertaken to evaluate the beneficial effects of the aqueous and methanol extracts of *A. hybridus* against CP-induced ovarian toxicity in rats.

## 2. Materials and Methods

### Plant collection and preparation of the aqueous and methanol extracts

*A. hybridus *leaves and flowers were harvested in February 2015 in Dschang (West Cameroon), and authenticated by Dr. Victor Nana at the Cameroon National Herbarium (voucher specimen N 42324/HNC). The leaves and flowers were shade-dried at room temperature and reduced into powder using an electric grinder. The resulting powder was used for the preparation of extracts.

To prepare the aqueous extract, *A. hybridus* powder (19 gr) was macerated in distilled water (250 ml) for 48 hr and filtered. The filtrate was oven-dried at 45 C for 2 days and 3.2 gr of residue was obtained, giving an extraction yield of 1.28%. The methanol extract was prepared by macerating 19 gr of *A. hybridus* powder in methanol (250 ml) for 72 hr. After filtration, the resulting filtrate was dried using a rotary evaporator (55 C, under reduced pressure) and 10.8 gr of residue was obtained (extraction yield: 4.32%).

### Animals

40 young female Wistar rats (10 wk, 170-200 gr) were used in this research. Rats were obtained from the animal house of the Department of Animal Biology, University of Dschang, Dschang, Cameroon. They were maintained in conditions suitable for animal welfare (22-25 C; 12 hr light/dark cycle). Each 1 kg of the rat chow consisted of soy flour (205.80 gr), cornflour (668.70 gr), fish meal (102.70 gr), cottonseed oil (1.10 gr), bone meal (10.30 gr), bio-multiple vitamin (1.1 gr) and sodium chloride (10.30 gr).

#### Estrus cycle monitoring and selection of rats with a regular estrous cycle

Twenty-five days prior to the treatment, vaginal smears were collected daily (9:00-10:00 AM) using a glass pipette. The smear was placed on a slide, fixed with methanol, stained with methylene blue (0.03%), dried, and examined microscopically with a 10 × objective (Zeiss, X10) (16). Cell descriptions were used to classify the rats based on the stages of their estrus cycle as reported previously (17). Animals with a normal (regular) estrus cycle for at least 3 consecutive cycles were selected for the experiment.

#### Animal grouping and treatment

40 rats with a regular estrus cycle were distributed into 8 groups (n = 5 each) as follows: group 1 (control), normal rats orally treated with distilled water (10 ml/kg); group 2 (CP+DW), rats co-treated with CP (EndoxanⓇ/50 mg) (5 mg/kg/day) and distilled water (10 ml/kg); group 3 (CP+TW 80), rats co-treated with CP (5 mg/kg/day) and 3% tween 80 (polysorbate 80) (10 ml/kg); group 4 (CP+CC), rats co-treated with CP (5 mg/kg/day) and clomiphene citrate (Clomid 50 mg) (2 mg/kg/day); groups 5-6, (CP+AE55; CP+AE110), rats co-treated with CP (5 mg/kg/day) and aqueous extract of *A. hybridus *at 55 mg/kg and 110 mg/kg, respectively; groups 7-8, (CP+ME55; CP+ME110), rats co-treated with CP (5 mg/kg/day) and methanol extract of *A. hybridus *at 55 mg/kg and 110 mg/kg, respectively.

All drugs were orally administered daily for 28 days. After examination of vaginal smears (from days 0-28), the frequency of occurrence of the estrus stage was determined using the following formula: Frequency (%) = (number of occurrence of anestrus stage/total duration of the observation) × 100

The percentage of cycle disruption was calculated according to the following formula: Percentage of cycle disruption = (number of unsettled cycles/total number of observed cycles) × 100

After 28 days of treatment, rats were euthanized by cervical dislocation under diazepam (Valuim roche, 10 mg/2 ml) (10 mg/kg) and ketamine (Panpharma, 5 ml) (50 mg/kg) anesthesia. Ovaries and uteri were removed, rinsed in 0.9% NaCl, and weighed. The right ovary was crushed for the assessment of E2 while the left ovary was preserved in Bouin's solution and used for histological studies.

#### Intra-ovarian estradiol concentrations and histology

From each animal, 1 ovary was used for ELISA and the contralateral for histology. After euthanasia, ovaries were crushed and homogenized in 0.9% NaCl (at 5%). The supernatant obtained after centrifugation (3000 × g for 10 min) of the homogenate was used to quantify E2 levels using an ELISA kit (Accubind, Monobind Inc. Lake Forest, USA) as carried out by Ndeingang et al. (17). Calibration of the curve was constructed after reading the absorbance at 450 nm. The absorbance was read within 5 sec. The standard for the determination of estradiol was prepared from standard stock solutions containing E2 at concentrations of 0, 10, 30, 300, and 1000 pg/ml (17).

Histological study of ovaries was done following a standard procedure. Briefly, samples were dehydrated in alcohol, embedded in paraffin, and sectioned. Ovary sections (5 μm thick) were stained with haematoxylin/eosin and microscopically examined using a light microscope (OLYMPUS, X200) at a magnification of x200. Mature follicles that contained antrum, oocytes, granulosa cells, and basement membrane were classified as normal (18).

### Ethical considerations 

This study was authorized by the Scientific Committee of the Department of Animal Biology, University of Dschang, Dschang, Cameroon, and was conducted following Standard Ethical Guidelines described in the European Economic Community guidelines, EEC Directive 2010/63/EU, of 22 September 2010 (19).

### Statistical analysis

Data were expressed as mean ± standard error of the mean (SEM). The differences between groups were analyzed by ANOVA, followed by Tukey's test using the STATISTICA software (version 8.0, StatSoft, Inc., Tulsa, USA). P ≤ 0.05 was deemed statistically significant.

## 3. Results

### Effects of the aqueous and methanol extracts of *A. hybridus* on the frequency of appearance of estrus stages 

A regular estrus cycle was observed in all females in the control group while CP-treated rats had a disturbed estrus cycle. The frequency of the estrus phase in the rats given clomiphene citrate was 93.55%. Oral administration of *A. hybridus* (55 and 110 mg/kg) extracts partially reversed the frequency of appearance of the different phases of the estrus cycle with a remarkable effect in rats administered with methanol extract at 110 mg/kg (Table I).

### Effects of the aqueous and methanol extracts of *A. hybridus* on the chronology of appearance of estrus stages 

The effects of the different treatmentson the chronology of the appearance of the estrus stages are presented in table II. All rats in the control group had 100% normal estrus cycles for 28 days. The percentage of disruption of the estrus cycle in the CP+DW and CP+TW groups was 94.14% compared to the control group (0%). In all rats administered with clomiphene citrate, the percentage of disruption was 100% because the estrus cycle was blocked at the estrus phase as indicated in table I. Interestingly, this disruption was partially prevented by the aqueous extract of* A. hybridus* (55 mg/kg, 25.71% of disruption; 110 mg/kg, 34.28% of disruption) and by the methanol extract of* A. hybridus* (22.86% of disruptionfor both doses)*. *So, overall,the efficacy of *A. hybridus *in preventingestrus cycle disruption was more effective in rats administered with the methanol extract (Table II).

### Effects of the aqueous and methanol extracts of *A. hybridus* on ovarian and uterine weights 

**Figure 1:**
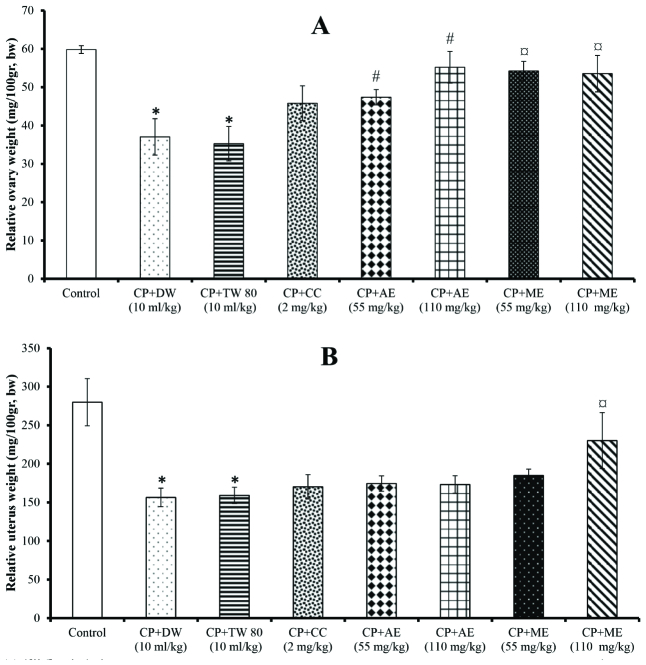
Effects of the aqueous and methanol extracts of *A. hybridus *on relative ovarian (A) and uterus (B) weights. Values are mean ± SEM. Number of animals per group = 5. DW: Distilled water, CP: Cyclophosphamide, TW: Tween, CC: Clomiphene citrate, AE: Aqueous extract, ME: Methanol extract. *P < 0.05: Significantly different compared with the control group. ${}^{\#}$ P < 0.05: Significantly different compared with the CP+DW group. P < 0.05: Significantly different compared with the CP+TW group. Magnification: 300 dpi.

The ovarian and uterine relative weights were significantly (p = 0.04) lowered after CP treatment, compared to the control (Figure 1). However, the aqueous and methanol extracts of * A. hybridus *significantly increased (p = 0.03) the ovarian relative weight compared to the CP+DW and CP+TW groups (Figure 1A). The uterine relative weight was also significantly (p = 0.04) elevated in rats given the methanol extract of *A. hybridus *at 110 mg/kg, compared with the CP+TW group (Figure 1B). The aqueous extract of* A. hybridus* (55 mg/kg) was more effective in modulating the ovarian and uterine relative weights (Figures 1A and 1B).

### Effects of the aqueous and methanol extracts of *A. hybridus* on E2 concentration

**Figure 2:**
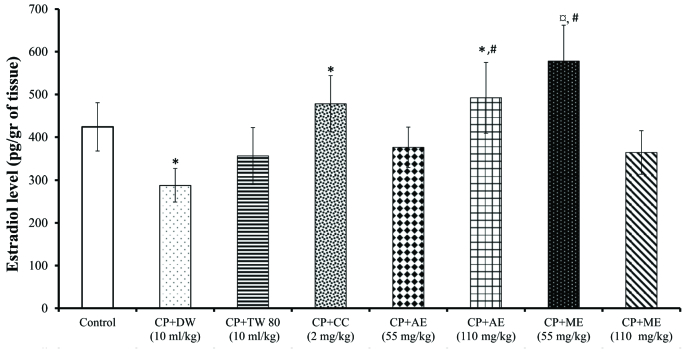
Effects of the aqueous and methanol extracts of *A. hybridus *on ovarian estradiol level. Values are mean ± SEM. Number of animals per group = 5. DW: Distilled water, CP: Cyclophosphamide, TW: Tween, CC: Clomiphene citrate, AE: Aqueous extract, ME: Methanol extract. *P < 0.05: Significantly different compared with the control group. ${}^{\#}$ P < 0.05: Significantly different compared with the CP+DW group. P < 0.05: Significantly different compared with the CP+TW group. Magnification: 300 dpi.

As shown in figure 2, CP treatment induced a significant (p = 0.03) decline in E2 levels, compared to the control group. This detrimental effect of CP on E2 concentration was corrected by clomiphene citrate (p = 0.02) and the *A. hybridus *extracts. Indeed, E2 levels were elevated by 40.16% (p = 0.05, CP+CC vs*.* CP+DW) in rats administered with clomiphene citrate, 41.66% (p = 0.04, CP+AE 110 mg/kg vs*. *CP+DW) in those administered aqueous extracts of *A. hybridus* (110 mg/kg), and 38.40% (p = 0.05, CP+ME 55 mg/kg vs*. *CP+DW) in those administered methanol extracts of *A. hybridus* (55 mg/kg). The methanol extract of *A. hybridus* (55 mg/kg) was more effective in improving ovarian E2 levels(Figure 2).

### Effects of the aqueous and methanol extracts of *A. hybridus* on ovarian histology

**Figure 3:**
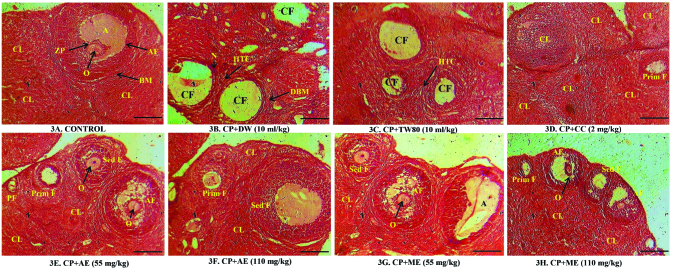
Effects of the aqueous and methanol extracts of *A. hybridus *on ovarian histology.Magnification: x40. Calibration bar = 100 µm. Magnification: 300 dpi, DW: Distilled water, CC: Clomiphene citrate, CP: Cyclophosphamide, AE: Aqueous extract, ME: Methanol extract, CL: Corpus luteum, CF: Cystic follicle, O: Oocyte, A: Antrum, ZP: Zona pellucida, BM: Basement membrane, PF: Primordial follicle, Prim F: Primary follicle, Sed F: Secondary follicle, AF: Antral follicle. HTC: Hyperplasia of theca cells, DBM: Destruction of the basement membrane.

In the control group, normal ovary architecture was visible. This was illustrated by the presence of the antrum, oocyte, zona pellucida, and a well-shaped basement membrane. A large number of corpus luteum was also observed (Figure 3A). On the contrary, CP caused severe damage in the ovary tissues. These alterations were characterized by the presence of many cystic follicles. Furthermore, there was hyperplasia of the theca cells, destruction of the basement membrane, and necrosis (Figures 3B and 3C). Interestingly, the detrimental effects of CP on the ovarian architecture were prevented by clomiphene citrate and the *A. hybridus *extracts (especially the methanol extract at 110 mg/kg). The ovaries of rats administered with the plant extracts had many corpus luteum confirming normal ovulation and normal follicles at various stages of development. Cystic follicles were absent (Figures 3D-3H).

Overall, the aqueous extract (55 mg/kg) of *A. hybridus *was more effective in improving the ovarian and uterine weights while the methanol extract (55 mg/kg) showed the highest effect on estradiol level. The highest improvement in frequency of appearance of estrus stages, chronology of appearance of estrus stages and ovarian histology was observed in rats treated with the methanol extract of *A. hybridus *at110 mg/kg.

**Table 1:** Effects of the aqueous and methanol extracts of *A. hybridus *on the frequency of appearance of estrus stages over 28 days


**Treatments **	**Proestrus**	**Estrus**	**Metestrus**	**Dioestrus**
**Control**	25.00	25.00	25.00	25.00
**CP + distilled water (10 ml/kg)**	7.86	6.48	42.77	42.89
**CP + tween 80 (10 ml/kg)**	5.00	12.00	41.02	41.98
**CP + clomiphene citrate (2 mg/kg)**	3.17	93.55	2.14	1.14
**CP + aqueous extract (55 mg/kg) **	20.71	22.86	27.14	29.29
**CP + aqueous extract (110 mg/kg) **	15.71	24.29	25.71	34.29
**CP + methanol extract (55 mg/kg) **	20.29	22.86	29.29	27.56
**CP + methanol extract (110 mg/kg) **	23.14	23.26	25.74	27.86
Data presented as percentages. CP: Cyclophosphamide				

**Table 2:** Effects of the aqueous and methanol extracts of *A. hybridus *on the chronology of appearance of estrus stages over 28 days


**Treatments **	**Normal cycles**	**Unsettled cycles**	**Percentage of disruption**
**Control**	7.0	0	0
**CP + distilled water (10 ml/kg)**	0.4	6.6	94.14
**CP + tween 80 (10 ml/kg)**	0.4	6.6	94.14
**CP + clomiphene citrate (2 mg/kg)**	0	7.0	100
**CP + aqueous extract (55 mg/kg) **	5.4	1.6	25.71
**CP + aqueous extract (110 mg/kg) **	4.6	2.4	34.28
**CP + methanol extract (55 mg/kg) **	5.4	1.6	22.86
**CP + methanol extract (110 mg/kg) **	5.4	1.6	22.86
CP: Cyclophosphamide			

## 4. Discussion

The main objective of this study was to investigate the preventive properties of aqueous and methanol extracts of *A. hybridus* on CP-induced ovarian toxicity in rats. CP is commonly used as an anticancer agent (20), but this chemotherapy is associated with various side effects, such as hemorrhagic cystitis, hair loss, bone-marrow toxicity, and reproductive toxicity (4). In addition, many authors have reported estrous cycle disorders, low E2 levels, and ovarian structural changes in the female rat after CP exposure (21). In the current study, CP treatment induced ovarian toxicity after 28 days. This was characterized by estrus cycle disruption, low ovarian and uterine weights, decreased ovarian E2 content, and severe damages to the ovarian architecture. These reproductive, hormonal, and architectural defects were prevented by the *A. hybridus *extracts.

CP has been widely used to induce ovarian toxicity in rats (10). After administration, CP is metabolized in the liver into acrolein (the active metabolite responsible for the side effects) that directly interacts with the granulosa cell DNA and forms covalent bonds with the nucleophilic substrates through its radicals (22). This inhibits DNA replication (6). This effect on DNA leads to severe damage of the germ cells in the ovaries and negatively affects the function of granulosa cells, thereby inhibiting estrogen synthesis and inducing oxidative stress (5). The ovarian toxicity observed after CP application in this study is similar to previous works (10).

Ovarian and uterine weights are important parameters used in the evaluation of female reproductive toxicity. In the current work, the significant decrease in ovarian and uterine weights following CP treatment may have been due to tissue lesions, marked atrophy, necrosis, and cell death, as reported by Sun and colleagues (23). Importantly, this decline in ovarian and uterine weights was prevented by the *A. hybridus *extracts. Similar results have been reported with the aqueous extract of *Citrus limonium *(24).

In a normal rat, the estrus cycle is under the control of several hormones, the major one being E2. This hormone is synthesized in granulosa cells from androgens under the influence of pituitary hormones, follicle-stimulating hormone, and luteinizing hormone. The estradiol level varies according to the phase of the estrus cycle (25). The estradiol level increases gradually from late diestrus to estrus and decreases from late estrus to metestrus. Since low ovarian E2 concentration results in ovarian growth failure and estrus cycle disorders (26), the disruption of the estrus cycle in rats administered with CP could be considered the result of decreased E2 concentration, as reported by Akunna et al. (24).

Moreover, toxicity related to anticancer drugs is associated with ROS over-production and oxidative stress (14). ROS negatively affect cellular lipids and DNA, leading to abnormal protein production, and inhibit steroidogenesis in ovarian cells. Thus, the estrus cycle disruption and low ovarian E2 content observed in the CP-treated rats may also have been due to oxidative stress. However, further studies are needed to clarify this action.

Our results are similar to those of Firas and Huda who showed that administration of CP to normal rats led to the disruption of the estrus cycle. In both studies, in all rats given clomiphene citrate, the estrus cycle was blocked at the estrus phase (21). Clomiphene citrate is an effective ovulation stimulator used in the treatment of anovulatory infertility (22). Clomiphene citrate acts by blocking the E2 receptors, resulting in a high synthesis of gonadotropin-releasing hormone, which in turn elevates the secretion of luteinizing hormone, causing ovulation (22). Estrus cycle blockage observed in rats administered with clomiphene citrate may be due to its ability to block E2 receptors beta in the pituitary axis and modulate follicular growth, as reported before (22). These results are similar to a previous report (27).

The *A. hybridus* extracts prevented the disruption of the chronology and frequency of the different phases of the estrous cycle after CP exposure. This preventive effect could have been due to the presence of various active components. In another study it was shown that tannins present in *A. hybridus* extracts possessed steroidogenic properties (28). In addition, phytochemical analysis of this plant has revealed the presence of rutin, quercitins and alkaloids, which could exhibit steroidogenic and antioxidant activities, as found previously (17, 29). In parallel, Akunna et al. showed that *Citrus limonium* improved the growth of ovarian follicles and prevented oxidative stress in an experimental model of ovarian cytotoxicity (24).

The detrimental effects of CP on ovarian architecture have been reported (10). In the current work, CP altered the ovarian morphology and induced an accumulation of cystic follicles, due to its toxic effects. Kocahan and colleagues reported that in their study, CP inhibited the development of the antral follicles by interacting with ovarian follicular growth and inhibiting estradiol production in rats (3). In addition, a low number of primordial and primary follicles, and an overproduction of atretic and cystic follicles in CP-exposed female rats have been noted by other research teams (10, 30). Of great interest, animals co-treated with plant extracts and CP showed normal ovarian structure (presence of normal follicles at different stages of their development), which may explain the high E2 levels observed in these groups. Overall, the methanol extract of *A.* *hybridus* was more effective than the aqueous extract. This may be due to the ability of methanol to easily extract active components (such as alkaloids, phenols, saponins, steroids, tannins, and triterpenes) (12), which may be the compounds responsible for the preventive effect of *A.* *hybridus*.

## 5. Conclusion

CP treatment induced ovarian toxicity, characterized by estrus cycle disruption, low ovarian and uterus weights as well as a decline in E2 levels and severe ovarian structural damages. These reproductive, hormonal, and structural alterations were prevented by *A. hybridus *extracts. These results could further support the traditional use of *A.* *hybridus* as a fertility agent.

##  Conflict of Interest

The authors declare that there is no conflict of interest.
